# Book Reviews

**Published:** 1802-06

**Authors:** 


### CRITICAL RETROSPECT

### OF

### MEDICAL AND PHYSICAL LITERATURE.

---

I. Thesaurus Medicaminum; *or a new Collection of Medical Prescriptions, distributed into twelve Classes, and accompanied with Pharmaceutical and Practical Remarks, exhibiting a View of the present State of the Materia Medica and Practice of Physic, both at home and abroad.* 2d. Edit. By a Member of the London College of Phyſicians. 8vo. pp. 412. London. Baldwin, 1794.

This firſt volume, though publiſhed ſo long ago, is noticed at preſent for the ſake of introducing the ſecond, which is juſt publiſhed. The twelve claſſes into which the Materia Medica is here diſtributed, are, 1. Evacuants, viz. Errhines, Sialagogues, Expectorants, Emetics, Cathartics, Diuretics, Diaphoretics, and Emmenagogues. 2. Emollients, viz. Relaxants, Diluents, and Demulcents. 3. Abſorbents. 4. Refrigerants. 5. Antiſeptics. 6. Aſtringents. 7. Tonics. 8. Stimulants. 9. Antiſpaſmodics. 10. Narcotics. 11. Anthelminthics. 12. Heteroclites.

The public opinion reſpecting this volume, is ſufficiently declared by its extenſive ſale; and therefore, we ſhall proceed to the preſent continuation.

2. *A Prac-*

2. *A Practical Synopsis of the* MATERIA MEDICA, Vol. 2d. Part. I. containing Six of the Claſſes, viz. 2. Emollients. 3. Abſorbents. 4. Refrigerants. 5. Antiſeptics. 6. Aſtringents. 7. Tonics. By the Author of the THESAURUS MEDICAMINUM. 8vo. pp. 149. London, 1802. Baldwin, Seely, &c.

The author informs the public, that the ſecond part of this volume, which will complete the work, is preparing for the preſs, and will be publiſhed in the courſe of the enſuing winter.

In an advertiſement, he ſays, "Since the publication of the firſt volume of this work, eight years have elapſed. The author will not detain the reader with explaining the cauſes of this interruption; but will rather aſſign the reaſons why he has thus ſent forth a part only of the ſecond volume, without waiting until the whole ſhould be ready for the preſs. He has been induced ſo to do by the conſideration, that if he had withheld this part longer, he ſhould have heightened the diſpleaſure already incurred with the purchaſers of the firſt volume, If his other engagements have prevented him from bringing the work to a concluſion now, one advantage will reſult from it; *viz.* more opportunity will be allowed for inveſtigating and determining the powers and uſes of ſome new and not yet fully tried remedies, belonging to the important Claſſes of Antiſpaſmodics and Narcotics. Theſe, with the Claſſes of Stimulants, Anthelminthics, and Heteroclites, together with ſome ſupplementary pages relative to the neceſſity of altering and amending the Materia Medica conformably to the progreſs and improvements in Natural Hiſtory, Chemiſtry, Phyſiology and Clinical Practice,—ſhowing that this branch of medical knowldege is capable of being raiſed to a pitch of conſideration far above that which it has hitherto attained—will make up the remaining part of this volume.

"As ſome perſons may be diſſatisfied with the ſuppreſſion of the Author's name, he wiſhes it to be underſtood that it is his intention to affix it to the concluding part of the work."

To each Claſs is prefixed a tabular view of its contents, including food as well as medicines, and arranged according to the three kingdoms of Nature.

As a ſpecimen, we ſhall take the fifth Claſs, *Antiseptics*.

"(1.) *From the Animal Kingdom.*

Ammonia *acetata*. *Acetated Ammonia. Water of Acetated Ammonia.*

Ammonia *muriata*. *Muriated Ammonia. Salt Ammoniac.*

"(2.) *From the Vegetable Kingdom.*

Acetum *diſtillatum*. *Diſtilled Vinegar.*

Acetum *aromaticum*. *Aromatic vinegar.*

Acetum *camphoratum*. *Camphorated vinegar.*

Acetum *nitrosum*. *Nitrous vinegar.*

Anthemi *nobilis*. *Chamomile.*

Ariſtolochia *Serpentaria*. *Virginia Snake-root.*

Arnica *montana*. *Leopard's bane.*

Artemiſia *Abrotanum*. *Southernwood.*

Artemiſia *Absinthium*. *Common Wormwood.*

Artemiſia

Artemesia *maritima.* *Sea Wormwood.*
Cinchona *officinalis.* *Peruvian Bark.*
Citrus *medica.* *The Lemon.*
Conium *maculatum.* *Hemlock.*
Daucus *Carota.* *The Carrot.*
Dorstenia *Contrayerva.* *Contrayerva.*
Hordeum *vulgare.* *Barley. Malt.*
Laurus *Camphora.* *Camphor.*
Humulus *Lupulus.* *The Hop.*
Myrrha. *Myrrh.*
Oxalis *Acetosella.* *Woodsorrel.*
Papaver *somniferum.* *The White Poppy.* *Opium.*
Pinus *Larix.* *The Larch-tree.* *Turpentine.*
Prunus *spinosa.* *The Sloe.*
Ribes *nigrum.* *The Black Currant.*
Ruta *graveolens.* *Rue.*

---

Carbo *lignarius.* *Charcoal.*
Gas acidum *carbonicum.* *Carbonic acid gas.* *Fixed air.*
Fermentum *cerevisiæ.* *Yeast.*
Cerevisia. *Malt liquor.*
Vinum. *Wine.*
Spiritus *vini.* *Spirit of Wine*

" (3.) *From the Mineral Kingdom.*

Aqua *frigida.* *Cold Water.*
Acidum *muriaticum.* *Muriatic Acid.*
Acidum *vitriolicum.* *Vitriolic Acid.*
Acidum *vitriolicum aromaticum.* *Aromatised vitriolic acid.*
Spiritus Ætheris vitriolici. *Spirit of vitriolic æther.*
Argilla *vitriolata.* *Vitriolated argill.* *Alum.*
Argentum *nitratum.* *Nitrated Silver.*
Cuprum *acetatum.* *Acetated Copper.*
Cuprum *vitriolatum.* *Vitriolated Copper.*
Natron *boracicatum.* *Borax.*

---

Acidum *nitricum* *Nitric Acid.*

### " CLASS V. ANTISEPTICS.

(1) *From the Animal Kingdom.*

" Ammonia *acetata.* (See Refrigerants.) This is frequently and advantageously employed in malignant fevers.

" Ammonia *muriata,* Ph. Lond. et Ed. (Sal Ammoniacus) Muriated Ammonia. Salt Ammoniac. A neutral Salt compounded of the Volatile alkali and muriatic acid. It is given by some practitioners internally in malignant fevers, in doses of ten or thirty grains, dissolved in the camphorated mixture, bitter infusions or decoction of cinchona; but it is more frequently used in gargles and external applications.

(2) *From the Vegetable Kingdom.*

" Acetum. (see Refrigerants) Vinegar. This may be used in the manner described in the preceding class. It is frequently added to gargles. It may also be advantageously combined with Aromatics, as in the instance of the *Acetum Aromaticum,* Ph. Ed. which is made by infusing rosemary, sage, lavender and cloves in vinegar. A tea-spoon full, diluted with water, may be given for a dose.—It is moreover used as an odorament.—In some of the foreign Pharmacopœias there is an *Acetum Camphoratum,* which is made by dissolving Camphor in Spirits of Wine and then adding it to the Vinegar. Dose, the same as that of the aromatic vinegar. It is in like manner used for smelling to. Vinegar boiled with honey to the consistency of a syrup (*oxymel*) is used in gargles and lotions.

—By

—By others it has been combined with nitre; and fuch a mixture or folution (*Acetum nitrofum*) is faid to be remarkably efficacious in the fcurvy. (*Patterson* on the Scurvy, 1795. *Rollo* on Diabetes, 1798.) It would feem that the vinegar is the principal agent, as nitre alone is not beneficial in this difeafe. (*Trotter's* Effays.) When vinegar is employed to fumigate fick rooms, it fhould be boiled in glazed earthen pipkins and carried about the bed, but not thrown upon hot bricks and coals, or heated metallic utenfils, by which it is decompounded.

" ANTHEMIS *nobilis*. (Chamœmelum. Herba et Flores.) Chamomile. (See Vol. I. p. 173.) This herb and its flowers are employed in decoctions for antifpetic fomentations and clyfters. It is an ingredient in the *Decoctum pro Fomento*, Ph. Lond. (formerly termed Fotus Communis) which is made by boiling an ounce of dried chamomile, dried wormwood, and dried fouthernwood, and half an ounce of dried bay-leaves, in fix pints of water. Two fpecies of Artemifia were furely not needful in this decoction. The wormwood (in a double proportion) without the fouthernwood, or the fouthernwood without the wormwood, would have fufficed. The *Decoctum pro enemate*, Ph. Lond. (formerly called Decoctum commune pro Clyftere) belongs to Emollients. The *Decoctum Chamæmeli*, Ph. Ed. (called alfo *Decoctum Commune*) is made by boiling one ounce of Chamomile flowers and half an ounce of carraway-feeds in five pints (pounds) of water. This is ufed clyfterwife in quantities of a pint or more, alone or with various additions.—For other remarks on this bitter vegetable and its preparations, fee Tonics.

" ARISTOLOCHIA *Serpentaria*. (Serpentaria Virginiana.) Virginia Snake-Root. (See vol. I. p. 245.) Decoctions and infufions of this root, either alone or in combination with the Peruvian bark, are frequently prefcribed in the advanced ftages of low and malignant fevers. The proportions and dofes have been already mentioned in the preceding Volume, under Diaphoretics; where alfo notice is taken of the ufes and dofes of the tincture.

" ARNICA *montana*. Leopard's-bane. See Stimulants.

" ARTEMISIA *Abrotanum*. Syngenefia. Polygamia æqualis. Compofitæ. Difcoideæ. Frutex. Southern parts of Europe (Abrotonum. Folia vel fummitates.) Southern-Wood. Decoctions of the tops of this bitter-aromatic fhrub are frequently ufed as fomentations in cafes of bad ulcers, and gangrenous affections. It is an ingredient in the *Decoctum pro Fomento*, Ph. Lond. as mentioned under the article chamomile.

" ARTEMISIA *Absinthium*. Clafs and Order as the preceding, (Abfinthium) Common Wormwood. Employed, like the fouthernwood, in antifeptic fomentations.

" ARTEMISIA *maritima*. Clafs and order as above. (Abfinthium maritimum.) Sea Wormwood. Ufed in the fame manner as the Common Wormwood; and is an ingredient in the *Decoctum pro Fomento*, Ph. Lond.—There is no ufe in fwelling the catalogue of the materia medica with two fpecies fo fimilar in their fenfible qualities and medicinal effects. One of the two, the artemifia abfinthium, finthium, or this fpecies, fhould be expunged. For other remarks on this vegetable and its preparations, fee Tonics and Anthelminthics.

" CINCHONA *officinalis.* Pentandria. Monogynia. Contortæ. Arbor. Peru. (Cortex Peruvianus.) Jefuit's bark. Peruvian bark. In petechial and other malignant fevers, in the putrid fore throat, and in gangrenous affections, the powder, decoction, and tincture of this bark, are given with good effect, joined with acids, port wine, camphor, and opium. It may alfo be adminiftered clyfterwife in thefe cafes, and particularly to children labouring under the confluent fmall pox accompanied with a typhoid condition of the fyftem. (Thefaur. Med. p. 184.) For an account of the preparations and dofes of this valuable article of the materia medica, fee Aftringents and Tonics, under which cafes its other and more general ufes will be noticed.

" CITRUS *medica.* (Limon.) The Lemon. (fee Refrigerants.) The acid juice of this fruit is given in conjunction with the camphorated-mixture, decoction of cinchona, and other antifeptics, in malignant fevers, the putrid fore throat and gangrenous affections. (Thefaur. Med. p. 174—176.)—Added to red port-wine and water, it forms a ufeful beverage (called *Negus*) in all fuch cafes. —The *Succus Limonis Spiffatus*, Ph. Lond. duly diluted with water, fupplies the place of the frefh juice.—Repeated trials have proved that the acid juice of the lemon is antifcorbutic in a very high degree. In confequence of the teftimonies produced in its favour by various phyficians and furgeons, it has become a ftanding remedy in our Navy. The recent juice alone, or fweetened with fugar (and in fome cafes mixed with port wine) may be given in the quantity of many ounces in the fpace of twenty-four hours. If it excites diarrhœa, aftringents and aromatics muft be joined with it. Where the frefh juice cannot be procured, the cryftallized citric acid (prepared by faturating the filtrated juice with chalk, and afterwards adding to the wafhed precipitate vitriolic acid, according to the proceffes of *Scheele* and *Dizé*.—See *Fourcroy* Syfteme des Connaiffances Chimiques, Tom. VII.) may be employed in its place. *Lind.* on the Scurvy, 1754. *Blane's* Difeafes of Seamen, 1789. *Trotter* on the Scurvy, 1792, and his Medicina Nautica, 1797 and 1799.

" CITRUS *Aurantium.* Clafs and order as above. (Aurantium hifpalenfe) The Seville Orange. The juice of this may be given in fcorbutic cafes in the fame manner as the juice of the lemon. For its Conferve, fee Tonics.

" LAURUS *Camphora.* (fee vol. I. p. 251.) Camphor. This refinous fubftance, whofe preparations and dofes have been already mentioned in the preceding Volume as above referred to, has been often fuccefsfully employed in petechial and other malignant fevers (*Riverius—Hoffman—Huxham—Pringle*) and in gangrenous affections; in which laft it is ufed externally as well as internally (*Collin* Obfervationes circa Morbos.) In thefe cafes it is combined with the Peruvian bark, wine and acids. (*Thefaur. Med.* p. 171—174.) It has alfo been adminiftered clyfterwife with the beft effect to children dren labouring under the confluent ſmall pox accompanied with a typhoid ſtate of the ſyſtem. *Buckner* de uſu Cort. Per cum Camphora remixt. in febribus ex putredine ortis. 1762.

" HUMULUS *Lupulus,* Diœcia. Pentandria. Scabridæ. Indigenous. (Lupulus Coni.) The Hop. Poultices made of the dried bloſſoms or cones, macerated in water, have lately been applied to ill-conditioned and gangrenous ulcers, with very good effect. *Trotter's* Med. Nautica, Vol. II. *Duncan's* Annals of Medicine, Vol. II.

" MYRRHA. (See vol. I. p. 147.) Myrrh. Solutions of this gum-reſin in ſpirit of wine (ſee *Tinctura Myrrhæ,* Vol. I. p. 294) are frequently employed in lotions for foul ulcers, and in gargles againſt the gangrenous ſore throat. In the laſt-mentioned caſes it's more volatile particles may be conveyed to the fauces along with the vapour of hot water or vinegar. *Theſaur, Med.* p. 183.

" OXALIS *Acetoſella.* Decandria. Pentagynia. Gruinales. Indigenous. (Lujula. Folia.) Woodſorrel. The juice of this plant abounds in a peculiar acid termed the Oxalic ; which, like the citric, is powerfully antiſcorbutic.

" The *Conſerva Lujulæ,* Ph. Lond. (Conſerve of Woodſorrel) is prepared by bruiſing the leaves in a marble mortar, and then beating them together with thrice their weight of double-refined ſugar. A tea-ſpoon full or more may be given occaſionally.—The expreſſed juice may be taken in the ſame manner as lemon-juice. What is termed the *eſſential Salt of Woodſorrel* (obtained from the expreſſed juice by filtration, evaporation and cryſtallization) is not pure oxalic acid, but an acidulous ſalt (like the cryſtals of tartar) compoſed of the vegetable alkali ſuperſaturated with the acid of ſorrel. Half an ounce of this ſalt diſſolved in fourteen or ſixteen ounces of hot water, duly ſweetened, forms when cold, a pleaſant and uſeful beverage in malignant fevers.—The pure oxalic acid, which is identical with the acid of ſugar, is not uſed in medicine. *Savary* de Sale Eſſentiali Acetoſellæ, 1773. *Fourcroy* Syſtême des Connoiſſances Chimiques, Tom. VII.

" CARBO *lignarius.* (Carbonium.) Charcoal. Carbon. Charcoal duly prepared from wood, and reduced to a fine powder, has been applied to foul ulcers and mortified parts with apparent advantage. The powder may be mixed up with boiled bread and milk, and applied in the form of a poultice. *Crell's* Chemical Journal (Engliſh Tranſlation) Vol. III. *Beddoes* on Factitious Airs; and *Simmons'* Med. Facts and Obſerv. Vol. VII.

" Gas ACIDUM *carbonicum.* Aër fixus. Gas Mephiticum. Carbonic Acid Gas. Fixed Air. Mephitic Air. This elaſtic fluid is obtained for medicinal purpoſes by pouring vitriolic acid upon chalk or marble. The gas thus extricated may be combined with water (pure or with additions) by means of an apparatus, in common uſe, invented by Nooth. Water thus impregnated with the carbonic acid gas is preſcribed with good effect to patients labouring under typhus and other malignant fevers. It may be drank in the ſame quantities as pure water.—Much of the efficacy cacy of the acidulous ſoda-water depends upon its ſaturation with this air; which is likewiſe a principal agent in the ſaline effervеſc-ing draughts (being extricated from the prepared kali on the ad-mixture of vinegar or lemon-juice) and in all fermented bottled liquors, when in a ſparkling ſtate. Beſides being taken in theſe ways into the ſtomach, it has alſo been frequently drawn into the lungs in certain diſeaſes of that organ, and particularly in the ad-vanced ſtage of phthiſis pulmonalis. (*Percival's* Eſſays, Vol. I.) In this diſorder, however, it has diſappointed expectation in ſome late trials. Externally it has been applied to foul and cancerous ulcers with temporary good effect. (*Ewart's* two Caſes of Cancer, 1794.) But even here it ſeems to act merely as a palliative. Whatever effi-cacy the Fermenting-Cataplaſm (*Theſaur. Med.* p. 180) poſſeſſes, it is wholly to be aſcribed to this gas, which is gradually evolved from it. See *Beddoes's* publications on Factitious Airs. Alſo, *Pearſon* on the different Kinds of Air, 1795.

" FERMENTUM *cereviſiæ*. Spuma cereviſiæ fermentantis. Flos cereviſiæ fermentantis. Yeaſt or Yeſt, Within theſe few years this ſubſtance has been cried up as an excellent remedy in malig-nant fevers. Various teſtimonies have been produced for and againſt its antiſeptic virtues. At preſent the evidence on both ſides is nearly equal; it is therefore a matter which muſt lie over for future deciſion. It is given internally in doſes of a table-ſpoon full, mixed with water, porter, or wine and water. [In the laſt caſe, how much may be owing to the Wine?] Externally it has been applied to foul ulcers in the form of a cataplaſm. It ſeems to promiſe more ſucceſs as an outward than as an inward remedy. Whatever may be its effects, they are to be aſcribed partly to the carbonic acid and partly to the bitter principle of the hop which it contains. See *Beddoes's* Conſiderations on Factitious Airs, and Medical and Phyſical Journal for 1800 and 1801.

" CEREVISIA. Malt liquor. (See Vol. I. p. 102) Freſh table beer, ſpruce beer, porter and bottled beer, are good antiſcorbu-tics, and are often adminiſtered in low and malignant fevers, with the beſt ſucceſs. It is only when they have too laxative an effect that their uſe in ſuch caſes becomes improper.

" VINUM. Wine. (See Vol. I. p. 104) In the advanced ſtage of typhus and other malignant fevers, and in mortificati-ons, Port Wine is perhaps the moſt powerful of all antiſeptics. In ſuch diſorders it may be given to the quantity of ſeveral pints within the ſpace of twenty-four hours.—Claret is preferable to port wine in many of theſe caſes.—Perry and Cyder, which may be conſidered as weaker ſorts of wine, may be employed for the ſame purpoſes.—In the fevers above-mentioned it is a proof that wine and other fermented liquors agree, if, during their uſe, the tongue becomes more moiſt, the ſkin more ſoft, the pulſe leſs frequent and more full, and the affection of the brain more mode-rate. Where the contrary effects are obſerved, they ſhould be di-miniſhed or diſcontinued.

" SPIRITUS *vini*. (Vol. I. p. 105) Spirit of Wine. Ar-dent Spirits. Brandy is ſometimes employed internally in the laſt

ſtage ftage of petechial fevers, gangrenous fore throats, and in the black-vomiting of the yellow-fever; in which laft it is perhaps more to be relied upon than any other medicine; but in the common low and malignant fevers of this country it is feldom adviseable to exhibit it otherwife than in a ftate of dilution with water and admixture with acids, as in the ftate of Punch; a liquor which may in fome meafure fupply the place of wine.—Externally fpirit of wine is employed, alone or combined with camphor, as an embrocation and fomentation in bruifes and mortification.

" (3) *From the Mineral Kingdom.*

" AQUA *frigida.* In malignant and peftilential fevers Cold Water, employed internally and externally, in the manner and with the cautions mentioned under Refrigerants, proves a moft powerful antifeptic. If faturated with the carbonic acid gas or mixed with vinegar, it is, in the opinion of fome practitioners, ftill more efficacious.

" ACIDUM *muriaticum.* Muriatic Acid. (Spiritus Salis Marini.) See Refrigerants. This acid, added to water in fuch quantities as render it pleafantly fharp to the tafte, affords an ufeful medicine in typhus and other malignant fevers; but we muft not imagine with a modern German phyfician, Profeffor *Reich*, that this and the other mineral acids are, without the aid of other agents, adequate to the cure of all fevers. They are even hurtful in fome conditions of fever. It may likewife be prefcribed in gargles in the cynanche gangrænofa. For thefe purpofes however does it poffefs any advantage over the vitriolic acid? (*Fordyce* on the Virtues of Muriatic Acid in the cure of putrid difeafes, 1789.) The muriatic acid vapour, extricated from fea-falt by pouring ftrong vitriolic acid upon it, is often employed for fumigating the apartments of thofe who have laboured under infectious fevers. The ozygenated muriatic vapour or oxy-muriatic vapour (obtained by mixing pulverized manganefe with the falt, before the vitriolic acid is added) anfwers ftill better for the fumigating procefs than the common acid vapour. See *Guyton-Morveau* in the Annales de Chimie, 1801, and *Rollo's* Account of the Artillery Hofpital at Woolwich, 1801.

" KALI *oxmuriatum.* Lixiva *oxmuriata.* Murias oxygenatus potaffæ. Oxymuriated kali. Oxymuriated lixiva. Oxygenated muriat of potafh. A falt compounded of the vegetable alkali and the oxygenated muriatic acid. It has lately been employed with good effect in cafes of typhus. Dofe from three to five grains. *Garnett* in *Duncan's* Annals of Medicine, Vol. II. and III. Alfo Medical and Phyfical Journal, 1801.

" ACIDUM *vitriolicum.* Vitriolic acid. (See Refrigerants) This acid is employed in malignant fevers, diluted with water, in the fame manner as the muriatic acid; but it is more frequently mixed with decoctions of cinchona, anguftura, contrayerva, &c. It is alfo employed in gargles. Dofe of the diluted acid (*Acidum vitriolicum dilutum*, Ph. Lond. twenty or forty drops.—The *Acidum vitrioli aromaticum* Ph. Ed. (formerly called Elixir vitrioli acidum)

 is is made by mixing (cautiously) six ounces of vitriolic acid with two pounds of rectified spirit of wine, and digesting in a gentle heat for three days; afterwards adding an ounce and a half of cinnamon, and one ounce of ginger, and digesting again for six days; then filtering. Dose thiry to sixty drops, in water, decoction of cinchona, &c.—*Spiritus Ætheris vitriolici,* Ph. Lond. et Ed. (formerly called *Spiritus vitrioli dulcis.*) see p. 43. Sixty or eighty drops of this dulcified acid may be given for a dose (joined with camphor or other aromatics) in malignant fevers. See *Carmichael Smyth* in Med. Communications, Vol. I. Also on the Jail Distemper, 1795, &c, &c."

